# Addressing Knowledge, Attitudes and Practices Toward Dengue Fever, Vector Control, and Vaccine Acceptance Among the General Population in Singapore

**DOI:** 10.3390/tropicalmed11030064

**Published:** 2026-02-26

**Authors:** Alicia X. Y. Ang, Po Ying Chia, Penny Oh

**Affiliations:** 1 Woodlands Hospital, National Healthcare Group, Singapore 737628, Singapore; 2 National Center for Infectious Diseases, Singapore 308442, Singapore; poying.chia@nhghealth.com.sg; 3 Department of Infectious Diseases, Tan Tock Seng Hospital, Singapore 308433, Singapore; 4 Lee Kong Chian School of Medicine, Nanyang Technological University, Singapore 308232, Singapore; 5 Takeda Pharmaceuticals (Asia Pacific) Pte Ltd., Singapore 018983, Singapore

**Keywords:** dengue, knowledge, attitudes, practices, vaccine, Singapore

## Abstract

Dengue remains a public health concern in Singapore, with endemic transmission and recurring outbreaks. This study presents results from a Singapore-focused subgroup of the Growth and Emerging Markets Knowledge, Attitudes, and Practices (GEMKAP) cross-sectional survey, which assessed public Knowledge, Attitudes, and Practices (KAP) levels related to dengue and prevention. A total of 400 adult respondents from Singapore participated in an online survey conducted between September and October 2022. Overall KAP scores were 48% (Knowledge), 61% (Attitudes), and 36% (Practices). Awareness of dengue transmission was widespread (96% identified mosquitoes as the vector and 97% recognised stagnant water breeding), while fewer respondents recognised the availability of a dengue vaccine (23%) or the absence of a medicinal cure (38%). Trust in the government’s dengue control efforts was high, though respondents practised an average of 5.1 out of 10 recommended prevention measures. Of the respondents, 25% had a high willingness to vaccinate against dengue. Multivariate analysis revealed that positive vaccine perceptions, past dengue experience, automatic motivation, and social opportunity were associated with willingness to vaccinate. Respondents supported a multi-pronged dengue management approach combining education, vector control, and vaccination. Future efforts should integrate behaviour change strategies, enhance multi-stakeholder collaboration, and empower communities to ensure sustainable impact.

## 1. Introduction

Dengue ranks among the most common vector-borne viral diseases worldwide. Its transmission has intensified in recent decades, with the World Health Organization classifying it as one of the top ten public health threats globally. Worldwide, dengue incidence, morbidity, and mortality continue to be on the rise [1]. In Singapore, dengue is endemic, marked by persistent year-round transmission and recurrent outbreaks. However, in comparison to other endemic countries in Asia, Singapore has a relatively low dengue force of infection [2,3].

Singapore has experienced dengue epidemics of increasing magnitude over recent decades [2]. In 2024, Singapore reported 13,610 dengue cases, a 36% increase from the 9949 cases recorded in 2023 [4,5]. Among these, 45 cases progressed to dengue haemorrhagic fever (DHF), a severe and potentially fatal complication of dengue infection [5]. Hospitalisation rates for dengue patients in Singapore range between 12% and 22% [6], with an average hospital stay of 3.8 days. This duration is notably longer for elderly patients (aged 65 and above), extending to 5 to 8 days on average [7]. Beyond its clinical toll, dengue imposes a substantial economic burden. From 2010 to 2020, the estimated national cost of dengue reached SGD 148 million, significantly higher than the SGD 58–110 million recorded in the preceding decade [7]. Notably, indirect costs such as productivity losses accounted for 21–63% of total dengue-related expenditures [7]. Beyond the clinical and economic burden attributed to acute dengue illness, there is also likely an additional burden from post-acute dengue infection complications. There is increasing recognition of complications, including cardiovascular and neurovascular complications, post-acute dengue infection, with associated chronic health loss and economic burden [8,9].

Recognising the burden of dengue, the Singaporean government has undertaken strong efforts in vector control. An Inter-Agency Dengue Task Force, comprising government agencies and town councils, has been set up to ensure that mosquito control measures are carried out and sustained [10]. Simultaneously, community engagement programmes, including the “Do The Mozzie Wipeout” campaign, have been rolled out since 2013 to encourage residents to play a part in the fight against dengue [11]. However, despite concerted efforts in public education and vector control, dengue outbreaks continue to emerge in a cyclical pattern every five to seven years, with the cycle length becoming shorter in recent years [12]. Hyperendemic dengue outbreaks were observed in Singapore in 2005, 2013, 2020 and 2022, potentially due to factors such as climate conditions, changes in dominant dengue virus serotypes and low herd immunity [13]. The National Environmental Agency (NEA) in Singapore has been reported to comment that “[the] population’s immunity to all four dengue virus serotypes remains low. The continued presence of all these dengue risk factors may lead to a potential surge in dengue cases, if insufficient action is taken” [4]. Dengue seroprevalence among Singaporean residents has declined substantially over the last five decades, a testament to the success of Singapore’s dengue vector control programme. Paradoxically, the resulting progressively low herd immunity increasingly exacerbates dengue outbreak risk [10].

The clinical management of dengue is also currently limited to supportive management, with no dengue-specific treatment available. A clinical trial on dengue antiviral EYU-688 has begun its Phase 2 trial, while another, JNJ-1802, has been discontinued [14]. As a consequence, treatment options for dengue are likely to remain supportive in the next few years [15].

Vaccination can be an effective way of protecting the at-risk population [16]. In Singapore, CYD-TDV is the only dengue vaccine currently approved, and its use is limited to individuals with a history of prior dengue infection and in a limited age range of 12 to 45 years of age; however, its production will soon be discontinued worldwide [17]. Other dengue vaccines, such as TAK-003, have not yet received approval in Singapore, and Butantan-DV remains in development.

This manuscript presents a Singapore-focused subgroup analysis of the GEMKAP study to better understand public perceptions of dengue, including attitudes toward vector control, vaccine confidence, and willingness to vaccinate. The study also seeks to assess current gaps and behavioural drivers specific to the Singapore context. This study aims to generate actionable insights and key messages for stakeholders involved in the design and implementation of effective, locally tailored dengue management programmes.

The Singapore-specific analysis was derived from a broader cross-sectional study (GEMKAP) that included Latin American (Argentina, Brazil, Colombia, and Mexico) and Asian (Indonesia, Malaysia, and Singapore) countries [18]. The cross-sectional GEMKAP study leveraged the Knowledge, Attitudes, and Practices (KAP) framework to evaluate individuals’ level of Knowledge of dengue disease and prevention; Attitudes toward the risk of infection and the effectiveness of vector control methods; and Attitudes and Practices related to community-based vector control, personal protective measures, and vaccination.

## 2. Materials and Methods

This analysis is derived from data collected by the wider GEMKAP study, which evaluated KAP relating to dengue disease, vector control, prevention, and vaccination among the general population in seven countries. To generate Singapore-specific insights, only Singapore data from the GEMKAP study were used, with no new data collected. To ensure the contextual relevance and accuracy of these insights with the current Singapore context, two local infectious disease experts from public health institutions (National Centre for Infectious Diseases and Woodlands Health) were consulted.

### 2.1. Study Design

The GEMKAP study was a quantitative, cross-sectional online survey conducted between September and October 2022. The study was conducted in accordance with the Checklist for Reporting Results of Internet E-Surveys (CHERRIES) to ensure the accuracy, validity, and reliability of the online survey methodology and data reporting [19]. For this analysis, only Singapore-specific data were extracted from the wider GEMKAP study dataset.

### 2.2. Participants

Potential survey respondents were recruited through an existing general-purpose web-based panel via email invitations sent through Kantar Profiles’ panel mailing list. Singapore contributed a sample size of 400 adult respondents, which was sufficient for descriptive analysis. All respondents completed the online survey voluntarily and provided consent to participate in the study. Those who completed the survey were provided with an incentive.

Eligible participants were between 21 (legal age in Singapore) and 60 years old. The upper age limit of 60 was set for two key reasons: First, dengue prevalence is higher among adults in endemic countries like Singapore, making prevention and management efforts more impactful in these groups. Secondly, limiting the survey to those 60 and younger helped to minimise variations in digital literacy among participants, thereby reducing potential selection bias. Quota sampling based on age, area of residence and household income, which were derived from the local census and publicly available data, was used to approximate the demographic profile of Singapore’s population. Respondents were recruited from all five regions of Singapore (Central, East, North, Northeast, and West), with regional quotas aligned proportionally to population distribution.

### 2.3. Survey Development

The GEMKAP study survey was developed by reviewing existing published dengue KAP studies. The survey captured information aligned with the KAP framework, covering several domains: understanding of dengue disease and its prevention methods, perceptions of infection risk and the effectiveness of vector control, behaviours related to community and personal dengue prevention, awareness, and attitudes toward dengue vaccination and rollout, practice methods of dengue prevention and preferred sources for health information retrieval. Two cognitive qualitative interviews were conducted in Singapore to refine, optimise, and validate the survey’s clarity, comprehension, decision-making, and response processes. The final survey comprised a total of 35 questions, which took approximately 30 min to complete. Responses elicited were of multiple formats—binary (true or false), Likert scale, multiple choice, and open-ended. Each question was categorised under a specific KAP subdomain.

Data validation measures were integrated into the online survey to ensure data quality and minimise entry errors. These included mandatory response fields, Internet Protocol (IP) verification, identity validation, digital fingerprinting, and engagement checks to identify irregular response patterns. Each participant was allowed to complete the survey only once, with duplicates excluded from analysis. After data collection, data cleaning was conducted to review the dataset by identifying and removing low-quality entries, including straight-line responses on multiple-choice questions, unusual patterns, atypical timestamps, and inconsistent or nonsensical answers in open-text responses.

### 2.4. Covariates and Outcomes

Sociodemographic variables collected in the GEMKAP study included gender, age, household size, ethnicity, religion, region of residence, level of education, and household income. Additional baseline characteristics gathered through the survey included dengue experience, perceived risk level (low, moderate, or high) of dengue, and vaccination history for dengue, COVID-19, and influenza. All data were self-reported by respondents. In this manuscript, APAC refers specifically to the aggregated data from Indonesia, Malaysia, and Singapore, while Global refers to the pooled dataset across all seven participating countries.

The primary outcome of the study was respondents’ willingness to vaccinate against dengue, which was measured using the Juster Scale, an 11-point numerical scale ranging from 0 (no chance) to 10 (certain). Scores of 8 to 10 indicated high willingness, 4 to 7 indicated moderate willingness, and 0 to 3 indicated low willingness to vaccinate.

Secondary outcomes focused on KAP regarding dengue infection, symptoms, prevention methods, and vaccines. Each survey question was assigned to a KAP subcategory, and composite scores were calculated for each subcategory and standardised on a 0–100% scale. Scores of 80–100% were classified as high, 50–79% as moderate, and 49% or below as low. These thresholds were based on established methodologies from previous KAP and dengue-related studies [20,21].

### 2.5. Data Analysis

Singapore-specific data were extracted from the GEMKAP study dataset for analysis using R software (version 4.2.1; R Foundation for Statistical Computing, Vienna, Austria). Descriptive statistics were used to report baseline characteristics such as sociodemographic factors, with percentages and counts for categorical variables and mean values and standard deviations for continuous variables. Descriptive analysis provides an overview of the data, key trends, frequencies, and central tendencies. In addition, subgroup analyses of secondary outcomes explored how KAP scores varied by factors including demographic characteristics, endemicity of residence, vaccine history, and overall vaccine attitudes. To identify Capability, Opportunity, and Motivation behavioural drivers that may influence vaccine uptake, multivariable regression using generalised linear models was employed.

### 2.6. Ethics and Data Confidentiality

The GEMKAP study received exemption status from the Pearl Institutional Review Board. Before participating in the study, each respondent provided informed consent electronically. No personal identification information was collected, stored, or transferred during the survey. All data were handled anonymously and in compliance with local privacy laws in the study countries. Data were securely stored and accessed with permission, analysed in aggregate, and archived via a permission-based access system.

## 3. Results

A total sample size of 400 respondents from Singapore was analysed from the wider GEMKAP study. Table 1 displays the sociodemographic characteristics of these study participants. The distribution of respondents across genders and age groups was uniformly balanced. More than half of the respondents lived in households of three or more persons (72%), while most had two or fewer children (94%). Most respondents held tertiary-level education (73%), and nearly half had a medium level of income (45%). Across respondents, 65% and 46% lived in highly endemic areas. Highly endemic regions were defined as those with moderate-to-high transmission levels (East, North, and West Singapore) relative to the rest of the country. Only 15% of respondents reported being previously infected by dengue.

While 93% of respondents were vaccinated against COVID-19, there was low uptake for other vaccinations, such as influenza (37%) and dengue (7%).

**Figure 1:**
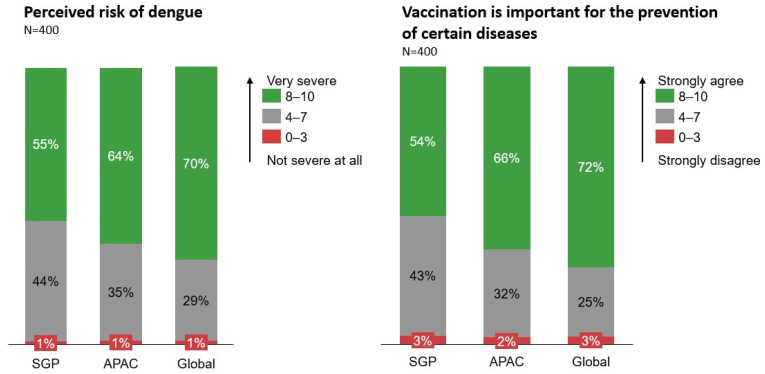
Perceived dengue risk and importance of vaccination.

About half (55%) of respondents recognised dengue as a severe disease (score of 8–10), which is comparatively lower than APAC (64%) and Global (69%) averages (Figure 1). A smaller proportion of Singaporeans (55%) perceived vaccines in general to be important for the prevention of certain diseases (score of 8–10), as compared to the APAC (66%) or Global (72%) average. Nevertheless, only a small percentage (1%) of Singaporeans perceived dengue to be not severe at all (score of 0–3), or that vaccinations are not important (score of 0–3) (3%).

### 3.1. Knowledge, Attitudes, and Practices

#### 3.1.1. Knowledge

**Figure 2:**
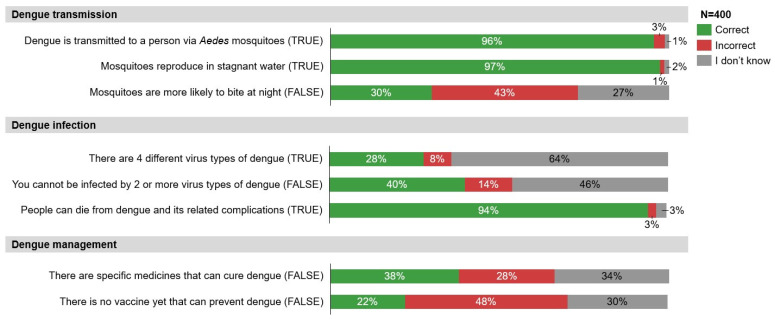
Knowledge of dengue transmission, infection, and management.

Dengue Knowledge levels in Singapore were similar (48%) to APAC (47%) and Global (48%) averages. In terms of dengue transmission and infection (Figure 2), there was a good understanding that dengue is transmitted via *Aedes* mosquitoes (96%), that mosquitoes reproduce in stagnant water (97%), and that people can die from dengue and its related complications (94%). However, there was low awareness of the four different dengue serotypes of the dengue virus (28%), that one can be infected by two or more virus types of dengue (40%), and that mosquitoes are not more likely to bite at night (30%). In terms of dengue management, only 38% of respondents were aware that there is no medicinal cure for dengue, and just 22% were aware that there is a vaccine to prevent dengue.

**Figure 3:**
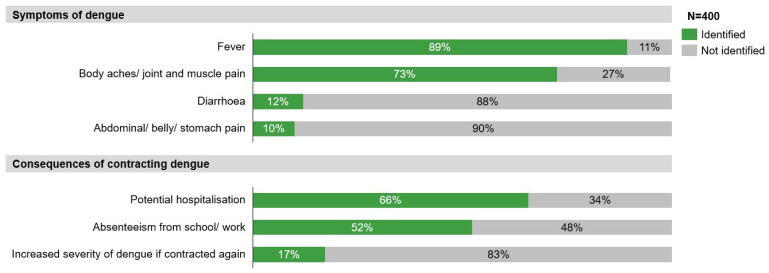
Knowledge of symptoms and consequences of dengue infection.

While there was good awareness of common symptoms of dengue, such as fever (89%) and body aches/joint and muscle pain (73%), only a few respondents recognised less-common symptoms such as diarrhoea (12%) and abdominal pain (10%) (Figure 3). Most respondents recognised the financial burden and clinical consequences of contracting dengue, such as potential hospitalisation (66%) and absenteeism from school/work (52%). However, less than a fifth knew the risks of dengue reinfection (17%).

#### 3.1.2. Attitudes

**Figure 4:**
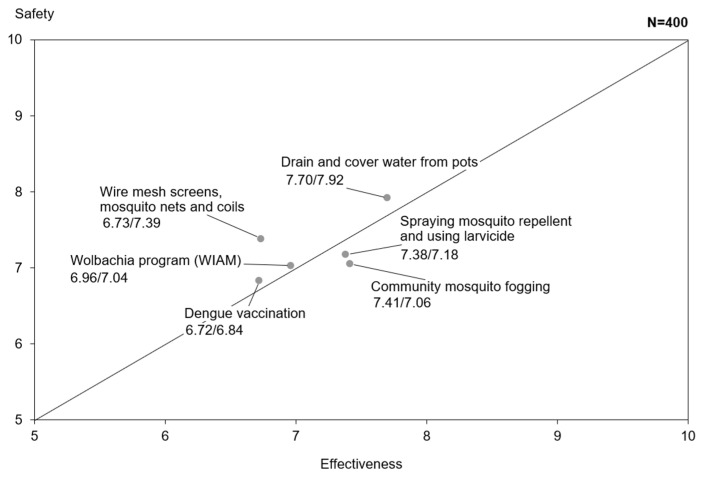
Perception of safety and effectiveness of dengue prevention methods. Each point represents the mean perceived safety (y-axis) and effectiveness (x-axis) rating. The diagonal line indicates equal perceived safety and effectiveness (y = x).

Attitudes scores were slightly lower (61%) among Singaporeans as compared to APAC (63%) and Global (66%) counterparts. Respondents in Singapore perceived the dengue prevention methods presented to be less safe and effective, as compared to APAC and Global respondents. Dengue vaccination ranked the lowest in both perceived safety and efficacy among all other methods (Figure 4).

Among all respondents, only 39% and 37% of respondents had high confidence (score 8–10) in the safety and effectiveness of a dengue vaccine, respectively. Almost half (46%) of respondents said they would wait to be reassured that there are no safety risks before vaccinating, and 35% did not want to be the first to try a new dengue vaccine. Nonetheless, only a small proportion of respondents did not believe in vaccines (8%) or thought that vaccines can cause autism (13%).

**Figure 5:**
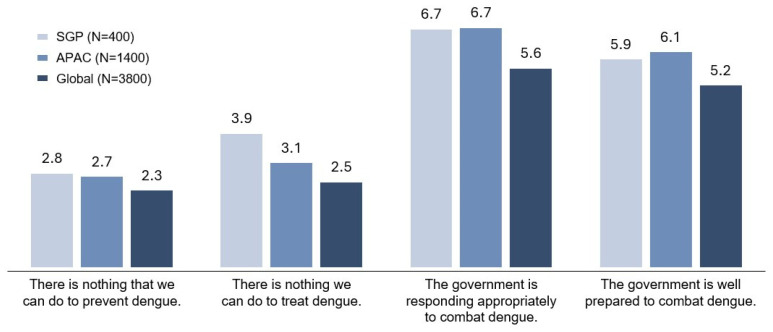
Level of agreement with statements on dengue management.

Statements presented as a negative sentiment, such as “there is nothing we can do to prevent dengue” (Singapore: 2.8, APAC: 2.7, Global: 2.3), “there is nothing we can do to treat dengue” (Singapore: 3.9, APAC: 3.1, Global: 2.5), ranked low in agreement (Figure 5). Additionally, Singaporeans had generally positive attitudes towards their government’s efforts in managing dengue. Statements such as “the government is responding appropriately to combat dengue” (Singapore: 6.7, APAC: 6.7, Global: 5.6) and “the government is well prepared to combat dengue” (Singapore: 5.9, APAC: 6.1, Global: 5.2) ranked higher in agreement as compared to Global averages and in line with APAC averages.

#### 3.1.3. Practices

Practice scores were lower in Singapore (SGP) (36%) relative to the Asia-Pacific (APAC) region (47%) and Global average (44%). Singaporeans practised, on average, 5.1 out of 10 dengue prevention measures presented (Table 2). Several methods were practised by fewer respondents in Singapore as compared to APAC and Global respondents, such as “throwing out open bodies of water” (SGP: 75%, APAC: 81%, Global: 81%), “adding larvicide in water containers to kill larvae” (SGP: 31%, APAC: 52%, Global: 39%), and “spraying insecticide and/or applying mosquito repellent” (SGP: 66%, APAC: 73%, Global: 70%). Other methods were practised by fewer respondents in Singapore as compared to APAC respondents, but slightly higher than Global respondents. These included “tightly covering all water containers” (SGP: 65%, APAC: 73%, Global: 63%), “keeping drains free of blockage” (SGP: 64%, APAC: 71%, Global: 62%), and “wearing long-sleeved shirts and/or long pants” (SGP: 38%, APAC: 41%, Global: 36%).

**Figure 6:**
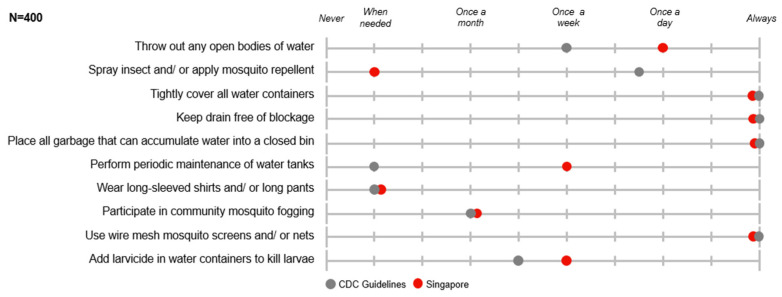
Frequency of prevention measures practised.

While Singaporeans practised a smaller range of dengue prevention measures on average, those measures practised met or surpassed frequencies recommended by the Centers for Disease Control and Prevention (CDC), except “spraying insect and/or applying mosquito repellent” (Figure 6).

### 3.2. Willingness to Vaccinate

**Figure 7:**
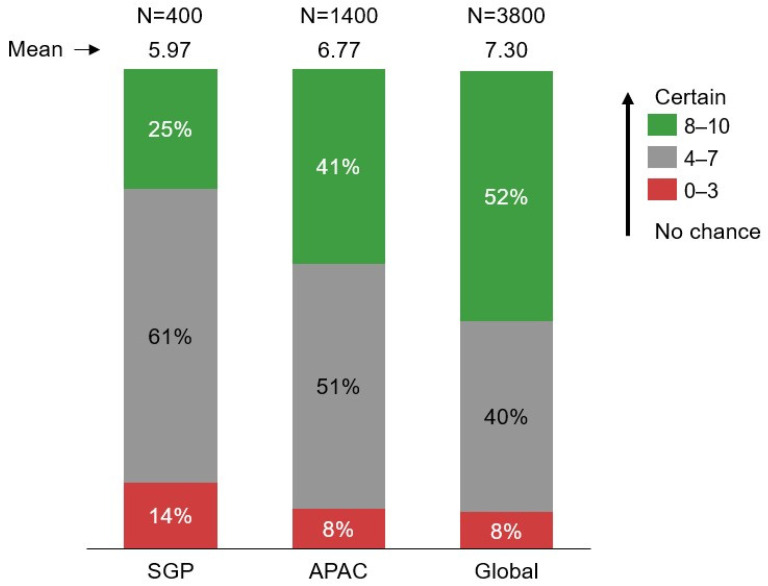
Willingness to vaccinate against dengue.

Singaporeans registered lower willingness to vaccinate against dengue and were less willing to recommend the vaccine to family and friends, as compared to APAC and Global respondents. Fewer Singaporean respondents (25%) had high willingness (score of 8–10) to vaccinate in comparison and APAC and Global, (APAC: 41%, Global: 53%) and more Singaporean respondents (14%) had low willingness (score of 0–3) to vaccinate (APAC: 8%, Global: 8%) (Figure 7). Additionally, the majority (>60%) of Singaporean respondents held a neutral position (score of 4–7) towards vaccinating themselves against dengue or recommending the vaccine to their family and friends.

**Figure 8:**
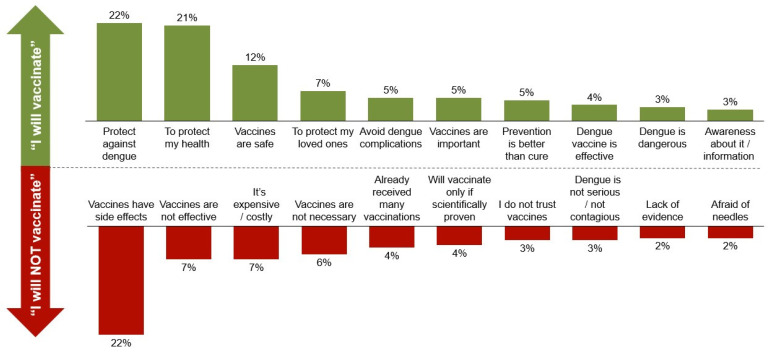
Reasons for willingness or unwillingness to vaccinate.

Subgroup analyses found that Singaporeans with past dengue experience had higher perceived risk of dengue, a positive opinion of vaccines, and those who were vaccinated for COVID-19 were more likely to have higher willingness to vaccinate against dengue. Additionally, respondents who perceived vaccines to be more useful were more willingness to vaccinate against dengue. Among respondents with higher willingness to vaccinate against dengue, key reasons cited were to protect against dengue (22%) and to protect their health (21%) (Figure 8). Conversely, subgroup analyses found that older individuals, those of lower income, had no children, perceived dengue to be of lower risk, and those who were not vaccinated against COVID-19 or influenza were more likely to have low willingness to vaccinate against dengue. Among respondents with lower willingness to vaccinate against dengue, the primary reason was concerns over vaccine safety (22%), which far exceeded subsequent reasons of vaccine ineffectiveness (7%) and cost (7%) (Figure 8).

The multivariate regression analysis for COM factors revealed that respondents with higher levels of automatic motivation and social opportunity were positively associated with willingness to vaccinate against dengue. Automatic motivation refers to the subconscious processes that drive behaviour, such as habits, impulses, and emotional responses. Statistically significant findings from the multivariable regression analysis indicated that, within the social opportunity domain, respondents who reported that doctors consistently recommended vaccines to their family members had a higher willingness to vaccinate against dengue. Within the automatic motivation domain, willingness to vaccinate was also higher among respondents who indicated that incentives, such as cash, points, or gifts, would encourage vaccination. Full multivariable regression results are provided in Supplementary Table S1.

### 3.3. Preferences in Communication and Dengue Management

In delivering health-related information, doctors (81%), followed by the government (66%), were rated as Singaporeans’ most trusted stakeholders. Preferred channels for health-related information were search engines (77%) and government or health agency websites (59%).

Religion was reported to have a limited impact on respondents’ health behaviours, with 17% agreeing that their religious beliefs guide their health decisions and that they live their lives according to their beliefs. The government and community leaders were found to have modest influence, with a quarter of respondents agreeing that the opinions of government and community leaders were important (24%). However, while there was agreement that their community organises events such as awareness campaigns and stagnant water clean-up events to promote good health (28%), only 18% actively participated in such events. Influencers had little influence on Singaporeans’ health-related events, with just 10% of respondents agreeing that the opinions of influencers were important.

**Figure 9:**
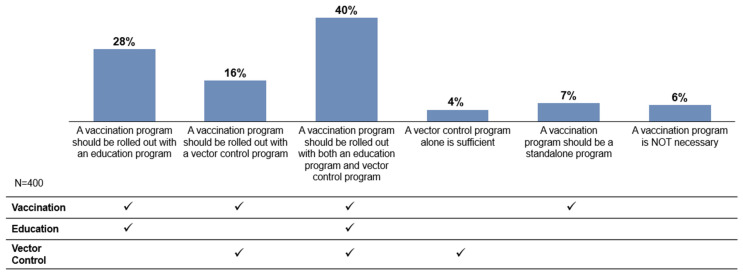
Preferred approach for dengue disease management. Check marks (✓) indicate the program components (vaccination, education, and/or vector control) included within each response option.

When asked about their preferred approach for dengue disease management, a three-pronged approach, which incorporated education, vector control, and vaccination (Figure 9), was most preferred. Fewer respondents preferred a two-pronged approach comprising vaccination and education (28%) or vaccination and vector control (16%). Only a minority believed that vaccination alone (7%) or vector control alone (4%) was sufficient.

## 4. Discussion

This cross-sectional study aimed to understand the Knowledge, Attitudes, and Practices of Singaporeans toward dengue disease and prevention, including willingness to vaccinate against dengue. The study found that Singaporeans’ dengue Knowledge scores generally matched regional and global averages, while Attitudes and Practices scores were relatively lower. Results also revealed Singaporeans’ lower willingness to vaccinate against dengue as compared to regional and global averages.

### 4.1. KAP Levels in Dengue Disease and Prevention

The analysis found that Singaporeans had moderate Knowledge about dengue, with strong awareness of dengue transmission methods and common symptoms. However, Knowledge gaps persist in key areas, such as the dengue serotypes, risk of reinfection, and the availability of a dengue vaccine. These findings align with a previous local KAP study, which highlighted gaps in public awareness of dengue prevention and misconceptions about preventive measures [22].

Singaporeans were generally optimistic that their government is well prepared and responding appropriately to combat dengue; however, individual responsibility and agency levels are comparatively lower, as compared to APAC and Global averages. A 2024 systematic review noted that dengue monitoring and management in Singapore is heavily driven by the authorities, reinforcing the perception that vector control is primarily a state responsibility, which may explain a high reliance on the government to manage dengue, rather than internalising it as an individual’s responsibility [7].

While some prevention methods in the survey, such as water tank maintenance or community fogging, may be less applicable to Singapore’s urban context, practice levels of locally promoted behaviours were also variable. These include actions from the national “Do The Mozzie Wipeout” campaign, such as the B-L-O-C-K steps (breaking up hardened soil, lifting and emptying flowerpot plates, overturning pails, changing water in vases, and keeping gutters clear using BTI insecticide) and the S-A-W steps (spraying insecticide, applying repellent, wearing long sleeves), encouraged in dengue cluster areas [11]. Several of the prevention methods outlined in these national programmes, such as removing stagnant water, applying larvicide, and using protective clothing, were practised less frequently among Singaporean respondents compared to APAC and Global averages. This discrepancy may reflect Singapore’s relatively low force of infection and declining seroprevalence, resulting in fewer individuals having personal exposure to dengue and therefore lower perceived vulnerability [2]. These findings point to opportunities to strengthen individual engagement and translate awareness into more consistent preventative behaviours.

A previous Singapore KAP study similarly found that while attitudes toward individual responsibility in dengue prevention were generally positive, with most respondents agreeing individuals have a role to play, actual engagement in specific preventative behaviours remained limited [22]. For example, only 25% reported regularly applying insect repellent, and 52% overturned or removed flowerpot plates [22]. This gap between intention and behaviour suggests an opportunity to support individuals in turning awareness into consistent action. Public engagement strategies that foster habit formation or provide practical tools, such as digital prompts or feedback mechanisms, could help reinforce these behaviours in daily life. Several factors may explain the lower practice scores. As dengue is well managed in Singapore through government interventions such as Project *Wolbachia*, the perceived urgency to take preventive action may be reduced [7,23]. High confidence in the healthcare system and the relatively low dengue mortality rate may also cause some to underestimate the potential severity of infection. As found in this study, misconceptions such as the belief that dengue has a medicinal cure and limited awareness of dengue as a vaccine-preventable disease may also contribute to a sense that prevention is less necessary or effective.

The analysis found that perceptions of vaccine usefulness and safety were less positive among Singaporeans than in other countries surveyed. This may reflect the narrow local indication of CYD-TDV, the only dengue vaccine currently available in Singapore. Dengue vaccination is only recommended for individuals aged 12–45 years with confirmed prior infection; the Ministry of Health (MOH) has explicitly stated that most people are ineligible and that CYD-TDV is not an effective tool for population-level control [24]. Familiarity with dengue vaccination in the general population is therefore limited, and public messaging has historically emphasised vector control rather than immunisation. These attitudes are also likely influenced by broader shifts in public views toward vaccination following the COVID-19 pandemic, which strengthened trust in vaccines for some groups but heightened hesitancy and safety sensitivity in others [25]. This evolving background context may have shaped Singaporeans’ perceptions of dengue vaccination at the time of the survey.

In this context, the study’s behavioural findings are notable. Positive vaccine attitudes were strongly associated with higher willingness to vaccinate. Notably, even marginal improvements in perceived usefulness, such as rating a vaccine “slightly useful,” were linked to increased willingness. Addressing safety concerns, which were the most frequently cited barrier to vaccination, will be critical. Public health messaging that highlights dengue severity, clarifies eligibility, and positions vaccination as a safe complementary measure to vector control may help strengthen acceptance.

### 4.2. Leveraging Behaviour Change Strategies

The comparatively higher Knowledge scores, alongside lower Attitudes and Practices scores, suggest that theoretical knowledge about dengue in Singapore may not be consistently translated into action. As Knowledge, Attitudes, and Practices factors are interconnected, greater understanding can foster more positive attitudes, which in turn can influence behaviour [26]. However, for this cycle to take effect, educational efforts must go beyond awareness to incorporate behaviour change strategies that reinforce habits. Campaigns such as “Do The Mozzie Wipeout,” which promote specific actions through the B-L-O-C-K framework, provide a foundation that can be strengthened by applying behavioural nudges and motivational triggers.

### 4.3. Addressing KAP Gaps Through a Multi-Pronged Dengue Management Programme

In developing a dengue management roadmap, a multi-pronged approach encompassing education, vector control, and vaccination was the most preferred approach by Singaporean respondents.

#### 4.3.1. Education

Evidence shows that educational strategies, particularly those that involve community participation, are effective in reducing *Aedes* breeding and dengue transmission [27]. Sustainable impact is more likely when interventions are tailored to the local context and involve co-development with community members to incorporate their feedback and needs [28]. In Singapore, education efforts should continue to highlight dengue severity and vaccine safety, which were two key gaps identified in this study. Messaging should be delivered through credible and trusted sources, such as doctors and government websites, and ensure that information found online is accurate and accessible.

#### 4.3.2. Vector Control

Singapore’s long-standing vector control programme has substantially reduced dengue transmission by at least tenfold since the 1960s to the 1990s [12]. Community involvement, supported by organisations such as the NEA, the People’s Association, and grassroots committees, has been central to its success [12]. However, the lower individual Practices scores suggest an opportunity to further emphasise the role of residents in sustaining these efforts. Reinforcing the message that vector control is a shared responsibility, and not solely a government task, may help address the perception that little can be done individually. A shift in mindset could encourage more consistent adoption of personal preventive measures.

#### 4.3.3. Vaccination

Vaccination complements vector control as a key additional source of prevention, especially for a country like Singapore, where herd immunity to the four dengue serotypes is low, increasing the risk for outbreaks [29]. Additionally, as the national vector control strategies, such as Project *Wolbachia*, are effective, the seroprevalence rate is declining [7,23]. This decline in population-level immunity, particularly among older adults with lower seroconversion and waning immune response, may heighten the risk of severe dengue outcomes, underscoring the importance of vaccination as a complementary preventive measure [30,31]. CYD-TDV is indicated only for seropositive patients and is being discontinued globally by its manufacturer due to low demand, and locally remains available only for individuals completing an ongoing three-dose series [32]. In contrast, newer vaccines such as TAK-003 (already approved in other markets) and additional vaccines currently in development are designed for use regardless of serostatus, expanding potential use to the general population [33]. Evidence suggests that dengue vaccination can substantially reduce infection rates, hospitalisations, and mortality, making it a critical complement to vector control and education in achieving long-term public health impact [34].

Beyond improving knowledge and attitudes, this study found that automatic motivation, such as cash incentives, and social opportunity, such as healthcare professional recommendations, are positively associated with willingness to vaccinate. These behavioural levers could be integrated into communication and delivery strategies to strengthen uptake over time.

### 4.4. Multi-Stakeholder Collaboration

The success of a multi-pronged dengue management programme hinges on strong collaboration among stakeholders, each bringing distinct expertise to design and implement effective strategies.

At the national level, policymakers and the Inter-Agency Dengue Task Force play a key role in setting and disseminating guidelines that inform local implementation. Healthcare professionals and government agencies are well-positioned to educate the public about dengue and prevention, as they are the most trusted sources to deliver healthcare-related information. Medical staff may be more effective in communicating treatment-related information, while other stakeholders, such as religious and community leaders, and social media platforms, can support broader awareness [35]. Education efforts should continue to highlight both the severity of dengue and the effectiveness of prevention, including the safety of vaccination.

Disseminating this information through preferred and credible channels, such as government and health agency websites, will help ensure wide and effective reach. Previous studies have also identified hospital websites, newspapers, television, and social media as common sources for dengue-related information due to their accessibility and trustworthiness [35]. Community and religious leaders can help foster a culture of health and serve as advocates for dengue prevention, especially when engaged in partnership with policymakers. Their involvement may help extend reach to segments of the population that are less responsive to traditional communication channels.

Finally, individuals can be encouraged to view dengue prevention not only as the government’s duty but also as a personal responsibility. Empowering communities to take ownership of their health can help strengthen both individual action and collective resilience against dengue in Singapore.

### 4.5. Strengths and Limitations

This subgroup analysis of the GEMKAP study provided more specific and contextualised insights into Singaporeans’ Knowledge, Attitudes, and Practices toward dengue disease and prevention, including willingness to vaccinate against dengue. The large cross-sectional sample achieved broad generalisability on KAP levels across Singapore while capturing a wide range of socio-demographic factors for a nuanced understanding of differences in knowledge, attitudes and practices.

There are several limitations to this analysis. Firstly, as the GEMKAP study relied on self-reported survey data, there is a risk of recall and social desirability bias. Secondly, while quotas were set for sociodemographic factors such as gender, age, and income to achieve national representativeness, other aspects, such as education level, were not set as a quota. Future KAP studies could consider incorporating additional sociodemographic quotas to further enhance the representativeness of the study sample. Thirdly, due to the cross-sectional nature of the study, causal relationships between willingness to be vaccinated and covariates cannot be established, with only associations being observed. In addition, the survey assessed general awareness of dengue vaccines and included questions on healthcare professional engagement (e.g., whether respondents received vaccination recommendations); it did not measure respondents’ detailed understanding of dengue vaccine mechanisms or mode of action, nor the extent or content of information communicated by healthcare providers. These elements may meaningfully influence vaccine acceptance and should be incorporated into future KAP studies. Finally, since data collection took place between September and October 2022, findings may not fully reflect current public perceptions, particularly in light of potential shifts in dengue incidence, prevention awareness, and the introduction of new dengue vaccines since then. At that time, public awareness and willingness to vaccinate were likely influenced by familiarity with the earlier dengue vaccine (CYD-TDV) available in Singapore, rather than newer vaccines that were still undergoing regulatory review globally. Future research should assess how knowledge and willingness to vaccinate may shift as awareness of newer vaccines increases. Finally, regularly conducting similar studies over time would enable more robust tracking of how knowledge, attitudes, and practices evolve alongside vaccine developments and dengue control initiatives.

## 5. Conclusions

This study found that while dengue-related knowledge among Singaporeans was moderate, attitudes and preventive practices were lower compared to regional and global counterparts. Strengthening all three domains may help improve willingness to vaccinate against dengue. A multi-pronged dengue management approach that combines education, vector control, and vaccination, preferred by most respondents, could be an effective framework for future efforts. To enhance dengue awareness, it is important to support the translation of knowledge into attitudes and actions through behaviour change strategies. While the government has implemented comprehensive vector control programmes, sustained individual engagement remains critical. Encouraging personal responsibility alongside public measures can help build community-wide resilience. Vaccination offers an important additional layer of protection, and by increasing vaccine confidence and uptake, this could reduce the health and economic burden of dengue over time. Achieving all of the above will require collaboration across stakeholders, including policymakers, healthcare professionals, and community leaders, to deliver consistent, trusted messages and foster collective responsibility in dengue prevention.

## Figures and Tables

**Table 1:** Socio-demographic characteristics of study respondents in Singapore.

Demographic	Sociodemographic	N (%), Total N = 400
Gender	Male	189 (47.25%)
	Female	211 (52.75%)
Age	21–30 years old	92 (23.00%)
	31–40 years old	103 (25.75%)
	41–50 years old	103 (25.75%)
	51–60 years old	102 (25.50%)
Household size	Live alone	30 (7.50%)
	1–2 members	83 (20.75%)
	3–4 members	210 (52.50%)
	5–6 members	67 (16.75%)
	>6 members	10 (2.50%)
Family household: children	No children	228 (57.00%)
	1–2 children	150 (37.50%)
	3–4 children	19 (4.75%)
	>4 children	3 (0.75%)
Ethnicity	Chinese	332 (83.00%)
	Indian	22 (5.50%)
	Malay	26 (6.50%)
	Others	20 (5.00%)
Religion	Buddhism or Taoism	160 (40.00%)
	Christianity	107 (26.75%)
	Islam	29 (7.25%)
	Hinduism	16 (4.00%)
	Others	8 (2.00%)
	No religion	80 (20.00%)
Education level	Primary education	3 (0.75%)
	Secondary education	57 (14.25%)
	Tertiary education	290 (72.50%)
	Post-tertiary education	50 (12.50%)
Level of income	High (≥12,500 SGD)	120 (30.00%)
	Medium (3000–12,499 SGD)	180 (45.00%)
	Low (<3000 SGD)	100 (25.00%)
Prior dengue infection ^1^	Yes	61 (15.25%)
	No	339 (84.75%)
Vaccinated against COVID-19	Yes	373 (93.25%)
	No	27 (6.75%)
Vaccinated against influenza	Yes	148 (37.00%)
	No	252 (63.00%)
Vaccinated against dengue ^2^	Yes	27 (6.75%)
	No	373 (93.25%)

^1^ Based on respondents’ perception, regardless of their actual dengue infection serostatus. ^2^ Based on respondents’ perception, regardless of their actual dengue vaccination status.

**Table 2:** Proportion of respondents practising dengue prevention measures.

Prevention Measures Practised	SGP (N = 400)	APAC (N = 1400)	Global (N = 3800)
Throw out any open bodies of water	75%	81%	81%
Spray insecticide and/or apply mosquito repellent	66%	73%	70%
Tightly cover all water containers	65%	73%	63%
Keep the drain free of blockage	64%	71%	62%
Place all garbage that can accumulate water into a closed bin	57%	67%	63%
Perform periodic maintenance of water tanks	43%	60%	63%
Wear long-sleeved shirts and/or long pants	38%	41%	36%
Participate in community mosquito fogging	36%	60%	53%
Use wire mesh mosquito screens and/or nets	33%	50%	49%
Add larvicide in water containers to kill larvae	31%	52%	39%
None of the above	5%	2%	3%
Average no. of prevention measures conducted (max. 10)	5.1	6.3	5.8

## Data Availability

The original contributions presented in this study are included in the article. Further inquiries can be directed to the corresponding author.

## Supplementary Materials

The following supporting information can be downloaded at: https://www.mdpi.com/article/10.3390/tropicalmed11030064/s1, Table S1: Multivariate Regression Results on Capability, Opportunity, and Motivation Associated with Willingness to Vaccinate against Dengue in Singapore.

## Abbreviations

The following abbreviations are used in this manuscript:

APAC	Asia-Pacific
CDC	Centers for Disease Control and Prevention
CHERRIES	Checklist for Reporting Results of Internet E-Surveys
COVID-19	Coronavirus disease 2019
DHF	Dengue haemorrhagic fever
GEMKAP	Growth and Emerging Markets Knowledge, Attitudes, and Practices
IRB	Institutional Review Board
KAP	Knowledge, Attitudes, and Practices
MOH	Ministry of Health (Singapore)
NEA	National Environment Agency
SGD	Singapore dollars
SGP	Singapore
WHO	World Health Organization
