# Supplementary material for: Addressing Knowledge, Attitudes and Practices Toward Dengue Fever, Vector Control, and Vaccine Acceptance Among the General Population in Singapore
Source: Trop Med Infect Dis. 2026 Feb 26;11(3):64. doi: 10.3390/tropicalmed11030064 (PMC13030492; doi:10.3390/tropicalmed11030064)
Supplement: Supplementary file 1 [file tropicalmed-11-00064-s001.zip › tropicalmed-4049740-supplementary.pdf]

SUPPLEMENTARY FILE

Supplementary Table S1: Multivariate Regression Results on Capability, Opportunity, and Motivation Associated with Willingness to Vaccinate against Dengue in Singapore

Statement	COEF	SE	P-value
**Capability Physical**			
"I am afraid of needles"	0.004	0.034	0.915
Number of activities below are you currently practiced	0.079	0.035	0.024
**Capability Psychological**			
"There is no vaccine yet that can prevent Dengue"	0.160	0.253	0.528
“How severe is dengue”	0.187	0.080	0.021
**Opportunity Environment**			
"The government has made it easy for people to get vaccinated by offering it at convenient locations"	-0.075	0.081	0.352
"It is easy to schedule a vaccination appointment"	0.183	0.082	0.026
"I believe that the vaccine should be made accessible to the public including myself"	0.248	0.075	0.001
**Opportunity Social**			
"The threat of dengue is or has been exaggerated by the media"	-0.015	0.051	0.775
"The threat of dengue is or has been exaggerated by the Government"	-0.029	0.045	0.522
"My doctor recommends my family and I vaccines for several health conditions as appropriate"	0.180	0.056	0.001
"I receive reminders from doctors/ the government about my upcoming vaccinations"	0.067	0.056	0.234
"The government has broadcasted education campaigns for people to get vaccinated"	-0.012	0.077	0.876
"My community/ government leader(s) (e.g. governors, mayors, councillors etc.) promotes the importance of vaccines"	0.001	0.073	0.990
"My favourite influencer(s) (e.g. local and international celebrities, etc.) promotes the importance of vaccines"	-0.017	0.051	0.735
"My community organizes events promoting health"	0.144	0.066	0.030
"My influencer(s) organizes events promoting good/ improved health and well-being"	0.022	0.046	0.626
**Motivation Automatic**			
"If I have to pay for the vaccination, I will not do it"	-0.090	0.041	0.029

"I will be more willing to get vaccinated if there are incentives (cash, points, or a gift)"	0.127	0.042	0.003
**Motivation Reflective**			
"Likelihood of people contracting dengue - Anyone"	0.251	0.187	0.181
"There is nothing we can do to treat dengue"	0.088	0.041	0.034
"There is nothing we can do to prevent dengue"	0.043	0.052	0.403
"We will all be completely powerless"	-0.054	0.055	0.323
"We just have to accept it"	-0.046	0.051	0.372
"I think vaccines are harmful"	0.021	0.048	0.669
"I trust the healthcare system and professionals in my country to deliver the vaccine and manage its side effects"	0.348	0.058	0.000
"If the risk of contracting dengue is low, I may not get the dengue vaccine"	-0.232	0.044	0.000
"My religious beliefs guide my health decisions"	-0.031	0.039	0.440
"The opinion of my community/ government leader(s) is important to me"	0.065	0.051	0.204
"The opinions of my influencer(s) is important to me"	0.122	0.044	0.006

COEF: Coefficient, SE: Standard Error
